# P39 Seroprevalence of HBsAg, HCV and HIV in community—an accidental detection in an emergency setup in a trauma centre, India

**DOI:** 10.1093/jacamr/dlaf230.046

**Published:** 2025-12-04

**Authors:** Madhavi Kirti, M Nizam Ahmed Parul Singh, Vanlal Tluanpuii, Bharat Chandra Das, Purva Mathur

**Affiliations:** Jai Prakash Narayan Apex Trauma Centre, All India Institute of Medical Sciences (AIIMS), New Delhi, India; Jai Prakash Narayan Apex Trauma Centre, All India Institute of Medical Sciences (AIIMS), New Delhi, India; Jai Prakash Narayan Apex Trauma Centre, All India Institute of Medical Sciences (AIIMS), New Delhi, India; Jai Prakash Narayan Apex Trauma Centre, All India Institute of Medical Sciences (AIIMS), New Delhi, India; Jai Prakash Narayan Apex Trauma Centre, All India Institute of Medical Sciences (AIIMS), New Delhi, India

## Abstract

**Objectives:**

HIV, hepatitis B and hepatitis C remain significant causes of morbidity and mortality in low resource settings. The Emergency department (ED)-based screening has proven effective in decreasing the spread of undiagnosed disease.

**Materials and methods:**

The spot test and ELFA(VIDAS) based tests for HBsAg, HCV and HIV was done for the patients who were brought to the trauma centre post traumatic injuries for treatment. These patients are otherwise healthy individuals but just the victims of accidents or any traumatic injuries.

**Results:**

The time period of study is from April 2019 to September 2024 i.e. 5 years and 5 months. The viral marker tests were done for the patients as screening and mandatory tests. Total samples received for HBsAg were 6976: 210 reactive (3%), 6766 non-reactive. Total samples received for HCV 6764:189 reactive (2.7%), 6575 non-reactive and total samples received for HIV were 6700: 118 reactive (1.7%), 6582 non-reactive.

**Conclusions:**

Since this was a mandatory test for viral markers so we can access the prevalence and also an accidental positivity of the infections in healthy people in the community. Higher seroprevalence is seen in accidently injured patients who are coming from community, hence there seems an unmet need of research in this field so that such individuals do not pose threat to themselves as well as community.

